# Trends in Beverage Consumption Among High School Students — United States, 2007–2015

**DOI:** 10.15585/mmwr.mm6604a5

**Published:** 2017-02-03

**Authors:** Gabrielle Miller, Caitlin Merlo, Zewditu Demissie, Sarah Sliwa, Sohyun Park

**Affiliations:** ^1^Division of Analysis, Research, and Practice Integration, National Center for Injury Prevention and Control, CDC; ^2^Division of Population Health, National Center for Chronic Disease Prevention and Health Promotion, CDC; ^3^Division of Adolescent and School Health, National Center for HIV/AIDS, Viral Hepatitis, STD, and TB Prevention, CDC; ^4^U.S. Public Health Service Commissioned Corps; ^5^Division of Nutrition Physical Activity, and Obesity, National Center for Chronic Disease Prevention and Health Promotion, CDC.

Beverages play an important role in the diets of adolescents because they help to maintain hydration and can provide important nutrients, such as calcium, vitamin D, and vitamin C (*1*). However, some beverages, such as sugar-sweetened beverages (SSBs) (e.g., soda or pop), provide calories with no beneficial nutrients. Beverage consumption patterns among American youth have changed over time; however, little is known about differences in consumption of various beverages by demographic characteristics such as grade in school, free/reduced price lunch eligibility, and race/ethnicity (*2*). CDC analyzed data from the 2007–2015 national Youth Risk Behavior Surveys (YRBS) to assess whether the prevalence of drinking non-diet soda or pop (soda), milk, and 100% fruit juice (juice) has significantly changed over time among U.S. high school students. During 2007–2015, daily soda consumption decreased significantly from 33.8% to 20.5%. During 2007–2011, daily milk and juice consumption did not significantly change, but during 2011–2015 daily milk and juice consumption decreased from 44.3% to 37.4% and from 27.2% to 21.6%, respectively. Although a decrease in daily soda consumption is a positive change, soda consumption remains high. Although there is not a specific recommendation for sugar-sweetened beverage consumption, the Dietary Guidelines for Americans 2015–2020 recommend that U.S. residents reduce sugar-sweetened beverage and sweet consumption to reduce intake of added sugars to less than 10% of calories per day. The Dietary Guidelines for Americans 2015–2020 recommend that persons choose beverages with no added sugars, such as water, in place of sugar-sweetened beverages, as one strategy for achieving the added sugars recommendation. Adolescents might need additional support in choosing more healthful beverages, such as low-fat milk, in place of SSBs.

The national YRBS is a biennial cross-sectional, school-based survey that provides representative data on health behaviors among students in grades 9–12 from public and private schools in the United States. In each survey, independent samples of students complete an anonymous, self-administered questionnaire during one class period and record their responses on a computer-scannable booklet or answer sheet. Participation by schools and students is voluntary. Study protocols are designed to protect students’ privacy. Detailed information about the national YRBS methodology has been described previously (*3*).

Questions about milk and juice consumption have been included on the national YRBS questionnaire since 1999; questions about soda consumption were added in 2007. Therefore, this analysis focuses on beverage consumption during 2007–2015 when sample sizes ranged from 13,583 to 16,410; overall response rates ranged from 60% to 71%.

Daily soda and juice consumption were assessed with the questions “During the past 7 days, how many times did you drink a can, bottle, or glass of soda or pop, such as Coke, Pepsi, or Sprite? (Do not count diet soda or diet pop)” and “During the past 7 days, how many times did you drink 100% fruit juices such as orange juice, apple juice, or grape juice? (Do not count punch, Kool-Aid, sports drinks, or other fruit-flavored drinks.)” Response options were “I did not drink soda or pop during the past 7 days” or “I did not drink 100% fruit juice during the past 7 days,” “1 to 3 times during the past 7 days,” “4 to 6 times during the past 7 days,” “1 time per day,” “2 times per day,” “3 times per day,” or “4 or more times per day.” Students who selected “1 time per day,” “2 times per day,” “3 times per day,” or “4 or more times per day” were categorized as daily soda or juice drinkers; all other students were categorized as non-daily soda or juice drinkers. Daily milk consumption was assessed with the question “During the past 7 days, how many glasses of milk did you drink? (Count the milk you drank in a glass or cup, from a carton, or with cereal. Count the half pint of milk served at school as equal to one glass.)” Response options were “I did not drink milk during the past 7 days,” “1 to 3 glasses during the past 7 days,” “4 to 6 glasses during the past 7 days,” “1 glass per day,” “2 glasses per day,” “3 glasses per day,” or “4 or more glasses per day.” Students who selected “1 glass per day,” “2 glasses per day,” “3 glasses per day,” or “4 or more glasses per day” were categorized as daily milk drinkers; all other students were categorized as non-daily milk drinkers.

Data from each survey were weighted to provide national estimates. Statistical software was used to account for the complex survey design of the YRBS. Prevalence estimates were computed overall and by school grade (9, 10, 11, 12), sex (male, female), and race/ethnicity (non-Hispanic white [white], non-Hispanic black [black], and Hispanic). Other and multiple racial/ethnic subgroups were excluded from the race/ethnicity subgroup analysis because the numbers were too small for meaningful analysis. Research indicates that income plays a role in the dietary choices of adults; however, little research has been done on the impact of socioeconomic factors on adolescents’ beverage choices (*4*). Therefore, a school-level variable, the percentage (low, middle, high*) of students in each school with free and reduced-price lunch (FRPL) eligibility, was assigned to each student record. Logistic regression analyses were used to assess linear and quadratic trends during 2007–2015 adjusting for grade, sex, race/ethnicity, and FRPL eligibility. When a significant quadratic trend was detected, the software Joinpoint^†^ was used to determine the year in which the trend changed direction or leveled off, known as the inflection point. Logistic regression models were then used again to assess the linear trends occurring in each segment (i.e., before and after the inflection point).

During 2007–2015, daily soda consumption decreased from 33.8% to 20.4%. During 2007–2011, daily milk and juice consumption did not change; however, during 2011–2015 daily milk and juice consumption decreased from 44.4% to 37.5% and from 28.2% to 21.6%, respectively (Table).

**TABLE:** Percentage of high school students who drink soda, milk, and juice daily by sex, grade, race/ethnicity, and free/reduced price lunch eligibility — National Youth Risk Behavior Surveys, United States, 2007–2015

Characteristic	2007	2009	2011	2013	2015	Linear change	Quadratic change 2007–2015*	
						2007–2015^§^	(2007–2011)	(2011–2015)
**Soda^†^**								
**Overall**	**33.8**	**29.2**	**27.8**	**27.0**	**20.4**	**Decreased**	**No change**	**No change**
**School grade**								
9	35.6	30.5	29.7	29.3	19.4	Decreased	Decreased	Decreased
10	33.2	29.2	27.3	25.4	20.8	Decreased	No change	No change
11	32.8	28.5	26.6	26.9	20.5	Decreased	No change	No change
12	33.1	28.3	27.0	26.0	21.0	Decreased	No change	No change
**Sex**								
Female	29.0	23.3	24.0	24.1	16.4	Decreased	No change	No change
Male	38.6	34.6	31.4	29.9	24.3	Decreased	No change	No change
**Race/Ethnicity**								
White, non-Hispanic	34.0	29.0	28.8	29.0	19.7	Decreased	No change	No change
Black, non-Hispanic	37.6	33.7	28.0	30.2	22.7	Decreased	No change	No change
Hispanic	33.4	28.1	27.0	22.6	21.7	Decreased	No change	No change
**School-level FRPL eligibility^§^**								
Low	27.0	24.3	24.9	21.0	15.6	Decreased	No change	Decreased
Mid	39.8	31.7	29.5	29.4	26.0	Decreased	No change	No change
High	38.3	37.8	35.4	33.2	24.5	Decreased	No change	No change
**Milk^¶^**								
**Overall**	43.1	43.9	44.4	40.3	37.5	Decreased	No change	Decreased
**School grade**								
9	45.4	45.9	46.8	42.1	38.6	Decreased	No change	Decreased
10	44.8	46.4	47.1	42.7	39.6	Decreased	No change	Decreased
11	40.3	41.7	42.5	37.5	35.8	Decreased	No change	Decreased
12	40.9	40.9	40.2	38.1	35.2	No change	No change	No change
**Sex**								
Female	35.0	34.2	34.8	31.7	28.2	Decreased	No change	Decreased
Male	51.1	52.8	53.4	49.0	46.2	Decreased	No change	Decreased
**Race/Ethnicity**								
White, non-Hispanic	47.8	49.9	48.8	44.5	41.2	Decreased	No change	Decreased
Black, non-Hispanic	28.1	26.0	29.0	26.2	25.1	No change	No change	No change
Hispanic	40.4	40.4	40.7	38.9	36.2	Decreased	No change	No change
**School-level FRPL eligibility^§^**								
Low	47.6	46.3	45.0	44.1	39.2	Decreased	No change	No change
Mid	41.5	41.3	43.4	38.8	34.3	Decreased	No change	Decreased
High	35.6	37.6	41.1	38.7	34.8	No change	No change	Decreased
**Juice^†^**								
**Overall**	**28.6**	**28.4**	**28.2**	**24.6**	**21.6**	**Decreased**	**No change**	**Decreased**
**School grade**								
9	29.4	29.1	27.7	25.1	22.5	Decreased	No change	Decreased
10	30.1	29.1	30.6	23.9	21.3	Decreased	No change	Decreased
11	26.6	27.4	27.4	25.5	21.9	Decreased	No change	Decreased
12	27.3	27.3	26.9	23.6	20.5	Decreased	No change	Decreased
**Sex**								
Female	24.3	24.3	23.9	20.9	17.7	Decreased	No change	Decreased
Male	32.7	32.0	32.2	28.3	25.3	Decreased	No change	Decreased
**Race/Ethnicity**								
White, non-Hispanic	25.6	26.9	26.3	21.0	19.0	Decreased	No change	Decreased
Black, non-Hispanic	35.0	33.3	33.2	32.8	27.6	Decreased	No change	Decreased
Hispanic	31.2	28.4	30.0	28.0	23.9	Decreased	No change	Decreased
**School-level FRPL eligibility^§^**								
Low	28.4	27.7	28.2	22.5	20.7	Decreased	No change	Decreased
Mid	27.4	29.0	26.5	26.3	20.1	Decreased	No change	Decreased
High	31.2	28.4	29.1	26.8	25.3	Decreased	No change	No change

**Abbreviation:** FRPL = free/reduced price lunch.

* Based on linear and quadratic trend analyses using logistic regression models controlling for grade, sex, race/ethnicity, and FRPL p <0.05.

^†^ Non-diet soda (soda) or 100% fruit juice (juice) one or more times per day.

^§^ The percentage of students eligible for enrollment in FRPL program in each school was divided into tertiles based on the overall distribution from http://www.schooldata.com/pdfs/MDR_Ed_catalog.pdf. FRPL categories were low = 0%–29%, medium = 30%–52%, and high = 53%–100%.

^¶^ One or more glasses of milk per day.

Among students in grade 9, daily soda consumption decreased significantly during 2007–2011 and then further decreased significantly during 2011–2015 (Table). Among students in schools with low FRPL eligibility, daily soda consumption did not change significantly during 2007–2011, but decreased significantly during 2011–2015. Across all other subgroups, daily soda consumption decreased significantly during 2007–2015. Among both female and male students; students in grades 9, 10, and 11; white students; and students in schools with middle and high FRPL eligibility, daily milk consumption did not change significantly during 2007–2011, then decreased significantly during 2011–2015. Among Hispanic students and students in schools with low FRPL eligibility, daily milk consumption decreased significantly during 2007–2015. Among students in grade 12 and black students, daily milk consumption did not significantly change during 2007–2015. Across all subgroups except one, daily juice consumption did not change significantly during 2007–2011 and then decreased significantly during 2011–2015. Among students in schools with high FRPL eligibility, daily juice consumption decreased significantly during 2007–2015.

## Discussion

Beverages contribute approximately 20% of calories to the diets of children and adolescents and can contain important nutrients (*2*). The 2015–2020 Dietary Guidelines for Americans recommend choosing beverages that are calorie free, (e.g., plain water) or that contribute beneficial nutrients (e.g., fat-free and low fat milk and 100% juice), instead of less nutritious options (*1*). The decline in milk consumption is a specific concern for adolescents because milk is a key source of calcium and vitamin D in the diets of persons in the United States; both are important for bone development, yet are under consumed (*1*).

Findings from this report and other recent studies indicate that adolescents are consuming less soda (*2*,*5*), which is encouraging because SSBs are one of the largest contributors of added sugars to adolescents’ diets (*6*). Several factors might be contributing to the decrease in soda consumption. First, new federal Smart Snacks in School^§^ nutrition standards were required at the beginning of the 2014–2015 school year, which eliminated sale of non-diet soda in high schools. Even before this requirement, many states and local school districts adopted policies limiting the sale of soda and other SSBs.^¶^^,^** In addition, community-based educational campaigns focused on reducing SSB consumption were implemented as recently as 2012 (e.g., Rethink Your Drink,^††^ Soda Free Summer^§§^). Despite these declines in soda consumption, intake of other SSBs, including energy drinks and sports drinks, are increasing (*2*,*5*), and overall consumption of all SSBs, such as soda, fruit drinks, and sweetened coffees and teas, remains high (*7*). Although no recommended amount on SSB intake exists, the goal should be to limit SSB intake to reduce added sugar. As an example, some childhood obesity prevention programs use a 5-2-1-0 message, which include no SSBs as the goal.^¶¶^ A recent analysis of consumption of all SSBs found that during 2011–2014, 62.9% of youth consumed at least one SSB on a given day accounting for 9.3% of total daily calorie intake for boys aged 12–19 years, and 9.7% of total daily calorie intake for girls aged 12–19 years (*7*). Therefore, policy and educational approaches (e.g., health education classes, community-wide campaigns) should continue to address SSBs, and promote healthier beverage options in multiple settings, including schools and communities.

Recent analysis of national data also indicates a decrease in juice consumption (*8*). Although fruit juice can provide important nutrients, including vitamin C and potassium, it is lower in fiber than whole fruit. Therefore, the 2015–2020 Dietary Guidelines for Americans emphasize primarily consuming whole fruit (*1*). Although most adolescents consume fewer than the recommended number of servings of fruit per day, they consume more whole fruit than 100% juice (*9*), and consumption of whole fruit has increased over time (*8*). In addition, only 10.2% of adolescents aged 14–19 years consume more than one 8-fl. oz. serving of juice per day (*9*). Multisector activities should continue to encourage youth to consume more whole fruit (*8*).***

The findings in this report are subject to at least three limitations. First, data are self-reported and might be subject to reporting and social desirability bias. A recent study showed that YRBS beverage questions underestimated the prevalence of daily non-diet soda intake but overestimated prevalence of daily milk and 100% juice intake compared with a 24-hour dietary recall interview (*10*). Second, these data apply only to adolescents who attend high school and are not representative of all persons in this age group. In 2012, approximately 3% of persons aged 16–17 years nationwide were not enrolled in a high school program and had not completed high school.^†††^Finally, trends in intake of other beverages frequently consumed by adolescents, such as water, could not be examined. Questions about water and sports drink consumption were added to the national YRBS questionnaire in 2015; questions about consumption of other SSBs, such as sweetened coffees and teas and fruit drinks, are not included at this time.

Multiple measures are needed to address adolescents’ beverage consumption and should reach settings where adolescents spend their time, such as homes, schools, and the community at large. Parents can influence the home nutrition environment through their food purchases (*11*). Schools can ensure that students have access only to healthier foods and beverages, provide opportunities for students to learn about healthy eating (e.g., nutrition education, taste tests), and use marketing and promotion strategies to encourage healthy choices.^§§§^ For example, schools can ensure students have access to free drinking water by having water fountains, dispensers, and hydration stations throughout the school, ensuring that water fountains are clean and properly maintained, and allowing students to have water bottles in class. Schools also can implement promotion campaigns to encourage students to drink water in place of SSBs.

Community-based strategies also should be considered. For example, health care providers can screen and counsel patients and their families on decreasing SSB intake, and organizations can implement social marketing campaigns to promote consumption of healthier beverages. Although the results of this report indicate a decline in soda consumption, there is a continued need to help adolescents shift beverage consumption patterns to more healthful options.

**Summary**

**What is already known about this topic?**

Beverages contribute approximately 20% of calories to the diets of children and adolescents and can contain important nutrients, but beverages can also contribute to excess consumption of added sugars and calories. Previous research has indicated that daily consumption of milk, juice, and non-diet soda has been decreasing over time, but little is known about trends among subgroups of youth.

**What is added by this report?**

During 2007–2015, daily soda consumption among U.S. high school students decreased significantly from 33.8% to 20.4%. During 2007–2011, daily milk and juice consumption did not significantly change, and then during 2011–2015 daily milk and juice consumption decreased significantly from 44.4% to 37.5% and from 28.2% to 21.6%, respectively.

**What are the implications for public health practice?**

Although the significant downward trends in daily soda consumption suggest that interventions encouraging reduced consumption of soda are working, overall prevalence of daily soda consumption remains high. Policy and educational approaches should continue to promote healthier beverage options in place of sugar-sweetened beverages.

## Footnotes

* The percentage of students eligible for enrollment in free and reduced-price lunch (FRPL) in each school was divided into tertiles based on the overall distribution from http://www.schooldata.com/pdfs/MDR_Ed_catalog.pdf. FRPL categories were low = 0%–29%, medium = 30%–52%, high = 53%–100%.

†
http://surveillance.cancer.gov/joinpoint/ .

§ National school lunch program and school breakfast program: nutrition standards for all foods sold in school as required by the Healthy, Hunger-Free Kids Act of 2010, 7 CFR Section 210 and 220. https://www.gpo.gov/fdsys/pkg/FR-2013-06-28/pdf/2013-15249.pdf.

¶
http://foods.bridgingthegapresearch.org/# .

**
http://www.bridgingthegapresearch.org/_asset/13s2jm/WP_2013_report.pdf.

††
https://www.cdph.ca.gov/programs/cpns/Pages/RethinkYourDrink.aspx .

§§
http://www.banpac.org/sugar_savvy_curr/banpac_soda_free_report_12_10_09.pdf .

¶¶
http://www.letsgo.org/

***
http://www.cdc.gov/vitalsigns/fruit-vegetables/index.html .

†††
http://nces.ed.gov/pubs2015/2015015.pdf .

§§§
https://wwwdev.cdc.gov/healthyschools/npao/pdf/mmwr-school-health-guidelines.pdf .
